# Lung cancer at a University Hospital in Saudi Arabia: A four-year prospective study of clinical, pathological, radiological, bronchoscopic, and biochemical parameters

**DOI:** 10.4103/1817-1737.58957

**Published:** 2010

**Authors:** Omer S. Alamoudi

**Affiliations:** *Department of Medicine, King Abdulaziz University Hospital, (KAUH), Jeddah, Saudi Arabia*

**Keywords:** Adenocarcinoma, lung cancer, large cell carcinoma, squamous cell carcinoma, small cell carcinoma, Saudi Arabia

## Abstract

**OBJECTIVES AND BACKGROUND:**

Lung cancer accounts for 4% of all newly diagnosed cancers in Saudi Arabia. The pattern of presentation is unknown. The objectives of this study were to assess the clinical, radiological, pathological, biochemical and bronchoscopic abnormalities in lung cancer patients and to compare our findings with those reported in the literature.

**METHODS:**

A total of 114 patients with proven lung cancer were selected for the study. A questionnaire concerning patients' demographic data was obtained; the abnormalities and the cell types of lung cancer were recorded prospectively in each subject.

**RESULTS:**

A total of 114 patients with lung cancer were studied. Mean age ± SD was (59.8 ± 10.8) years, and (71.1%) were smokers and 95.1% of them were male, (90.1%) smoked >20 pack/yr (96.2%) for 20 years or more. Cough (76.3%) and clubbing (40.4%) were the most common symptom and physical abnormality respectively. The right lung (64.9%) was more commonly affected than the left (37.7%). Metastases were present in (49.1%) at presentation. The right and left upper bronchi (24% vs. 16%) were the mostly affected. Hypercalcemia was more common in squamous cell, while hyponatremia was more common in adenocarcinoma, and small cell. Squamous cell carcinoma was the most common cell type (51.8%) and significantly associated with smoking (*P* ≤ 0.001)

**CONCLUSION:**

Squamous cell carcinoma was the most common cell type, and significantly associated with smoking. The incidence of metastasis was high at presentation. The right lung and right upper bronchus were often affected. Hypercalcemia and hyponatremia were the most common biochemical abnormalities.

Lung cancer is becoming a leading cause of death both worldwide and within the Kingdom of Saudi Arabia (KSA).[1–9] It accounts for 1.2 million new cases annually.[3] In the United States, during 2006, an estimated 170,000 were diagnosed with and over 160,000 died from lung cancer.[6] In the UK lung cancer is still among the most frequently occurring cancers and accounts for one in five of new cancer cases; that is 34,000 new patients annually.[10,11] In KSA, in contrast to early studies where lung cancer was rarely reported,[12–21] the prevalence of lung cancer has increased significantly in the recent years; this is, mainly attributed to the increased incidence of cigarette smoking among men and women in our community.[22] In 2004, the National Cancer Registry in KSA reported that lung cancer ranked fifth in males and seventeenth in females. There were 454 cases of lung cancer accounting for (4%) of all 11,330 newly diagnosed cancer cases and adenocarcinoma was the most common histopathology (29.7%) found.[7] In a recent study published from our centre; lung cancer was the 4^th^ leading cause of hospitalization among patients admitted with respiratory diseases.[8] Though the incidence of lung cancer is increasing, there was no previous study to assess the pattern of lung cancer in our patients. Therefore, the objectives of this study were, to assess prospectively the clinical presentations, radiological findings, pathological types, biochemical and bronchoscopic abnormalities in patients with histologically proven lung cancer, and to compare our findings with those reported in the literatures.

## Methods

This is a four year prospective study that was performed from October 2004 to September 2008 at King Abdulaziz University Hospital (KAUH), Jeddah, Saudi Arabia. Ethical approval of the study was granted from the Internal Review Board at KAUH. All participants gave written informed consent. A total of 140 consecutive patients with highly suspicious primary lung cancer detected clinically and radiologically were included for the study. The initial work up to diagnose lung cancer was either through performing flexible fiberoptic bronchoscopy (FFB), and/or computed tomography (CT)-guided biopsy. FFB was used primarily to diagnose central lung lesions while CT-guided biopsy was performed mainly to diagnose peripheral ones. Open lung biopsy was performed if bronchoscopy and/or CT guided biopsy had failed to obtain a diagnostic tissue. Mediastinoscopy and or pleural biopsy were performed when indicated to reach diagnosis. Total body scan of the bones and CT scan of the brain were used to assess the presence of metastasis. After the initial work up, a total of 26 patients were excluded from the study, 24 due to the benign nature of their illness and two withdrew from the study. A total of 114 patients with proven histopathology of primary lung cancer were interviewed, and clinically examined by the principle investigator (P.I.). A questionnaire concerning patient's demographic data, and smoking status as in Table 1 was filled out for each patient. Symptoms, signs and clinical examination related to primary lung cancer as in Table 2 were recorded. Chest radiographs, and CT scans of the chest, upper abdomen and brain, and the bone scan for each patient have been reviewed by a senior radiologist in our institution, the associated abnormalities and the distribution of the metastasis as in Tables 3 and 4 were recorded respectively. The diagnostic procedures used to reach the diagnosis of lung cancer were detected in Table 5. During bronchoscopy, all the abnormalities observed in the bronchial tree [Table 6] related to the primary lung cancer was recorded during procedure by the P.I. Brushing, washing and biopsy of the affected area was performed in all patients. The histopathology obtained (4–6 specimens/patient), and the cytology was independently examined by two senior pathologists in our institution, and the diagnosis of lung cancer was reached by consensus. The pathological diagnosis of primary lung cancer was in accordance with the revised World Health Organization (WHO) classifications of lung tumors.[23,24] Patients were distributed into four classes: squamous cell carcinoma, adeno carcinoma, large cell carcinoma, and small cell carcinoma. Measurement of serum calcium (Ca), sodium (Na), potassium (K), albumin, hemoglobin (Hb), was performed in each patient with lung cancer.

**Table 1:** Baseline characteristics and social demographics of patients (n = 114) with lung cancer

Characteristics	Findings	
Age (mean ± SD) years	59.8 ± 10.84	
Sex		
Males	89	78.1
Females	25	21.9
Nationality		
Saudi	74	64.9
Non Saudi	40	35.1
History of cigarette smoking		
Smokers	81	71.1
Mild = 10-19/pack/yr	8	9.9
Moderate = 20-36/pack/yr	30	37.0
Heavy ≥ 36 pack/yr	43	53.1
Non-smokers	33	28.9
Ex-smoker	00	00
Duration of smoking (in years)		
1-10	3	3.7
11-20	13	16.0
>20	65	80.2
History of shisha and cigarette smoking	9	7.9
History of shisha only	2	1.8
Duration of shisha (in years)		
1-10	3	27.3
>10-20	2	18.2
>20	6	54.5
History of tuberculosis	6	5.3
History of diabetes	22	19.3

**Table 2:** Symptoms, signs and chest examinations in patients with lung cancer

Clinical findings	No.	%
Symptoms		
Cough	87	76.3
Dyspnea	77	67.5
Weight loss	56	49.1
Anorexia	43	37.7
Hemoptysis	41	36.0
Chest pain	40	35.1
Fever	28	24.6
Orthopnea	18	15.8
Bone pains	13	11.4
Hoarseness	10	8.8
Constipation	9	7.9
Muscle weakness	8	7.0
Right hypochondria pain	7	6.1
Headache	6	5.3
Wheezes	3	2.6
Confusion	3	2.6
Signs		
Clubbing	46	40.4
Pallor	36	31.6
Jaundice	16	14.0
Cyanosis	14	12.3
Palmar erythema	13	11.4
Nicotine staining	11	9.6
Wasting small muscle of the hands	9	7.9
Hepatomegaly	8	7.0
Subcutaneous nodules	5	4.4
Superior vena cava obstruction	4	3.5
Gynecomastia	3	2.6
Deep vein thrombosis	3	2.6
Acanthosis nigricans	3	2.6
Horner's syndrome	2	1.8
Ascitis	1	0.9
Inferior vena cava obstruction	1	0.9
Herpes zoster	1	0.9
Chest examination		
Consolidation	41	36.0
Pleural effusion	38	33.3
Atelectasis	16	14.0
Normal	32	28.1

**Table 3:** Chest radiological findings in patients with lung cancer (n = 114)

Findings	No.	%
Affected lungs and lobes		
Rt. lung	74	64.9
Rt. upper lobe	44	59.5
Rt. middle lobe	16	21.6
Rt. lower lobe	20	27.0
Lt. lung	43	37.7
Lt. upper lobe	32	74.4
Lingula	8	18.6
Lt. lower lobe	12	27.9
Chest radiograph		
Lung mass	79	69.3
Pleural effusion	40	35.1
Atelectasis	31	27.2
Consolidation	26	22.8
Mediastinal lymph nodes	21	18.4
Hilar lymph nodes	15	13.2
Rib fractures and erosion	5	4.4
Lymphangitic carcinomatosis	5	4.4
Paralysis of the diaphragm	4	3.5
Cavity	3	2.6
Pleural thickening	3	2.6
Chest CT scan		
Lung mass	94	82.5
Mediastinal lymph nodes	58	50.9
Pleural effusion	41	36.0
Atelectasis	38	33.3
Hilar lymph nodes	34	29.8
Consolidation	31	27.2
Subcarinal lymph nodes	29	25.4
Paratracheal lymph nodes	23	20.2
Rib fractures and erosion	8	7.0
Lymphangitic carcinomatosis	7	6.1
Cavity	5	4.4
Pericardial effusion	2	1.8
Paralysis of the diaphragm	2	1.8
Radionuclide bone scans		
Positive for metastasis	56	49.1
Negative for metastasis	58	50.9

**Table 4:** Distribution of the metastasis sites among lung cancer patients (n = 114) at presentation

Site of metastasis	No.	%
Bones	56	49.1
Mediastinal lymph nodes	44	38.6
Hilar lymph nodes	35	30.7
Liver	34	29.8
Lung	34	29.8
Adrenals	10	8.8
Cervical lymph nodes	9	7.9
Brain	5	4.4
Skin	5	4.4

**Table 5:** Percentage of patients diagnosed as lung cancer (n = 114) using any of the different diagnostic procedures

Procedure	No.	%
Bronchoscopy and biopsy	68	59.6
CT guided true cut biopsy	39	34.2
Mediastinoscopy and biopsy	9	7.9
Open lung biopsy	5	4.4
Pleural biopsy	2	1.8
Skin	5	4.4

**Table 6:** Sites of the lung cancer and the other abnormalities during bronchoscopy (n = 68)

Sites of abnormal findings during bronchoscopy	No.	%
Vocal cord paralysis	13	19.1
Tracheal stenosis	3	4.4
Tracheal tumor	1	1.5
Broad carina	16	23.5
Right main bronchus	10	14.7
Right upper bronchus	16	23.5
Right middle bronchus	8	11.7
Right lower bronchus	6	8.8
Left main bronchus	11	16.2
Left upper bronchus	11	16.2
Lingula	8	11.8
Left lower bronchus	4	5.9

### Data management and statistical analysis

Data management was carried out using statistical package for social science SPSS 10 (Chicago, IL, USA).[25] Descriptive statistics (means, standard deviations, and frequencies) were performed to describe the studied variables. Chi-square test was used for cross tabulation. Level of significance was set at *P* value <0.05 throughout analysis.

## Results

**Figure 1:**
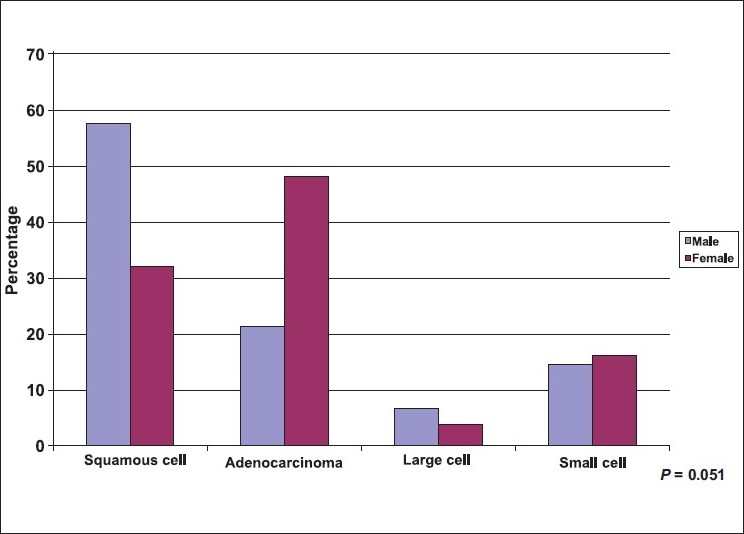
Distribution of lung cancer patients by histopathology and sex

**Figure 2:**
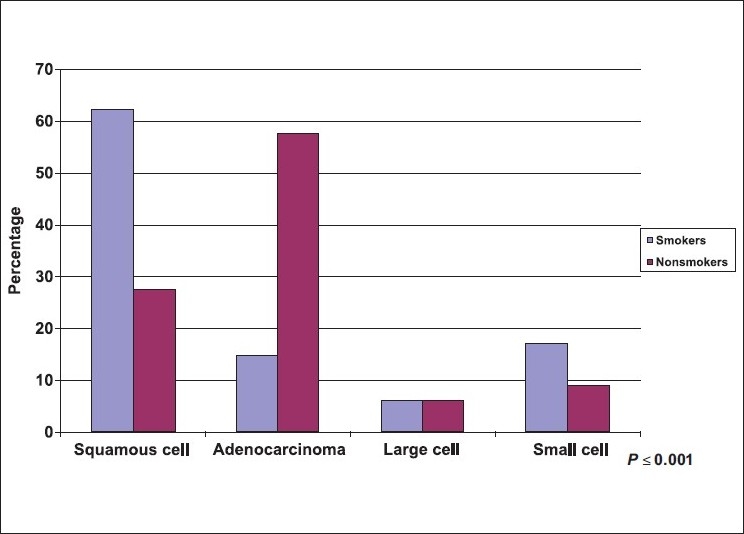
Distribution of lung cancer patients by histopathology and smoking

**Figure 3:**
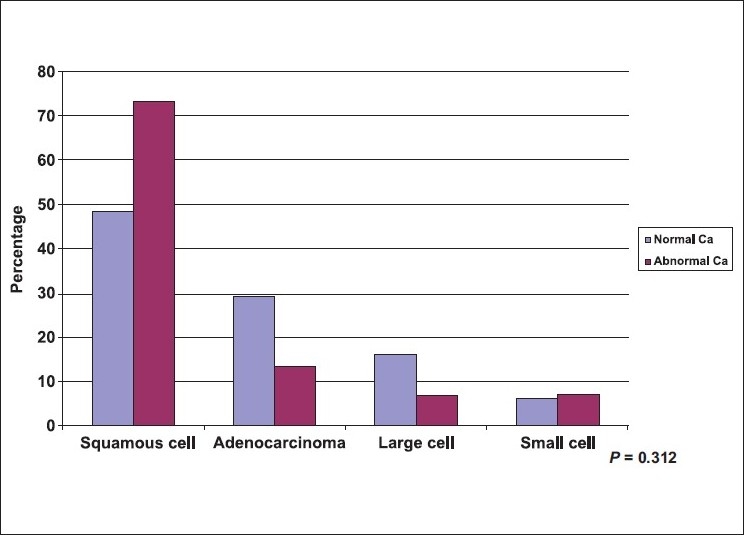
Distribution of lung cancer patients by histopatholgy and calcium (Ca) level

**Figure 4:**
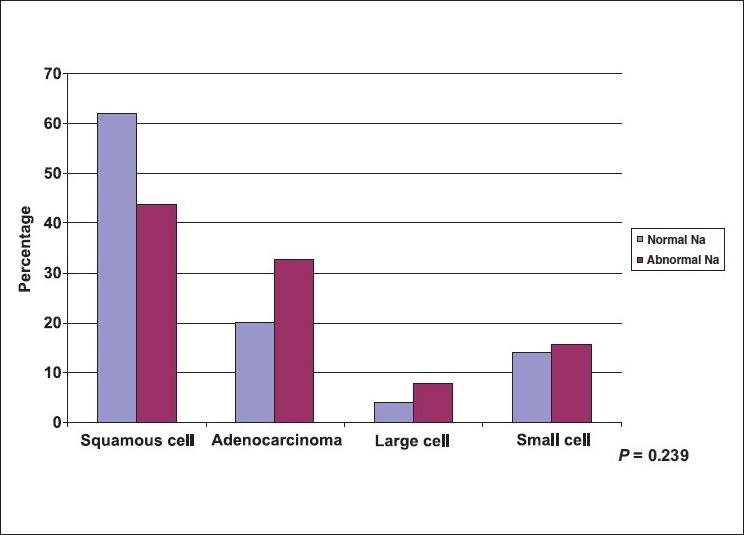
Distribution of lung cancer patients by histopatholgy and sodium (Na) level

A total of 114 lung cancer patients were studied, 78.1% were males. Mean age ± SD was (59.8±10.84) with a minimum age of 42 years and maximum of 80 years. A great proportion (71.1%) of patients were cigarette smokers, (95.1%) were male and more than half of them were heavy smokers >36 pack/yr. Less than 10% of the patients were shisha smokers (hubbly bubbly) [Table 1]. Table 2 shows the clinical characteristics of lung cancer patients. The most frequent symptoms were cough (76.3%), and dyspnea (67.5%). The most frequently observed physical signs were clubbing of the fingers (40.4%), and pallor (31.6%). Table 3 shows the chest radiological findings. The right lung was affected more frequently (64.9%) than the left lung (37.7%). In both lungs the upper lobes were commonly affected (59.5%) and (74.4%) respectively. The most frequent abnormal chest X ray findings were lung mass (69.3%), and pleural effusion (35.1%). As to the CT scan findings, lung mass was the most frequent finding (82.5%) followed by mediastinal lymph nodes (50.9%). Bone scan was positive for bony metastasis in (49.1%) at the time of presentation [Table 4]. The two most commonly used procedures to reach a diagnosis of lung cancer were FFB and biopsy (59.6%) and CT guided biopsy (34.2%), [Table 5]. The most frequent sites showing abnormal findings were right upper bronchus (23.5%), and carina, (23.5%) [Table 6]. The histopathological pattern among patients with lung cancer is shown in Table 7. Squamous cell carcinoma was the most common (51.8%), followed by adenocarcinoma (27.2%). Squamous cell carcinoma (57.3%) was significantly high among males as compared to females (32.0%), *P* = 0.051, while adenocarcinoma showed a higher frequency among females (48.0%) as compared to males (21.3%), [Figure 1], *P* = 0.051. Clearly Squamous cell carcinoma was significantly associated with smoking as compared with adenocarcinoma, *P* ≤ 0.001 [Figure 2]. Table 8, shows the biochemical abnormalities in lung cancer patients. Hypercalcemia was observed more frequently among squamous cell type compared to other types, *P* = 0.312 [Figure 3]. In contrast, hyponatremia was observed more among adenocarcinoma, and small cell as compared to squamous cell although it was not statistically significant, *P* = 0.239 [Figure 4].

**Table 7:** Histopathology and cytology of the lung cancer (n = 114)

Type	No.	%
Histology		
Squamous cell carcinoma	59	51.8
Adenocarcinoma	31	27.2
Small cell carcinoma	17	14.9
Large cell carcinoma	7	6.1
Cytology		
Squamous cell carcinoma	42	36.8
Adenocarcinoma	26	22.8
Small cell carcinoma	11	9.6
Large cell carcinoma	6	5.3
Negative cytology	29	25.5

**Table 8:** Biochemical abnormalities in patients with lung cancer (n = 114)

Abnormalities	No.	%
Anemia (Hb < 12 g/l)	77	67.5
Hypoalbuminemia (Albumin < 30 g/l)	66	57.9
Hyponatremia (Na < 130 mmol/l)	64	56.1
Hypokalemia (K < 3.5 mmol/l)	41	36.3
Hypercalcemia (Ca > 2.6 mmol/l)	15	13.2

## Discussion

The main findings were as follow: i) The most common presenting symptoms of lung cancer was cough (76.3%) followed by dyspnea (67.5%). ii) The most common physical signs were: clubbing of the fingers (40.4%) and pallor. iii) The right lung (64.9%) was affected more often than the left (37.7%), and lung mass (69.3%) and pleural effusion (35.1%) were the most common radiological abnormalities. iv) Almost 50% of the patients had distant metastasis at the time of diagnosis. V) Squamous cell carcinoma was the most common (51.8%) cell type followed by adenocarcinoma (27.2%). The clinical presentations of lung cancer result from the effects of local growth of the tumor, regional growth or spread through the lymphatic system, hematogenous distant metastatic spread, and remote paraneoplastic effects from tumor products or immune cross-reaction with tumor antigens.[26–27] In this study, similarly to previous ones,[26,28–30], cough (76.3%) was the most common presenting symptoms in our patients. The mechanism of cough was diverse and may be due to local growth in a central location, or less commonly in peripheral ones or it may be a feature of large airway obstruction causing postobstructive pneumonia, or caused by lymph nodes enlargement.[26,28] Dyspnea develops early in up to 60% of patients with lung cancer. It may occur due to either occlusion of a major airway, development of pleural effusion, lymphatic obstruction, phrenic nerve paralysis with an elevated hemidiaphragm, compromised lung disease, or by involvement of the heart and pericardium.[26,28–30] In this study, dyspnea developed in up to 68% of patients - a finding that was slightly higher than that previously reported in the literature of 60%. This may be due to a high incidence of advanced lung cancer at presentation. In our patients, the frequency of hemoptysis was identical to previous reports accounting for one third of patients with lung cancer.[26,28–30] Chest pain occurs in 60% of patient at diagnosis in previous studies. It often occurs as a result of either infection or infiltration of the pleural surface by the tumor, or due to rib metastasis, or direct invasion of the ribs or vertebrae by the tumor.[26,28–30] In our patients, for unknown reasons, the complaint of chest pain was slightly less common than previously reported occurring in only 35%. However, the high incidence of diabetes in our patients may partly explain this. Clubbing of the fingers is a common sign of lung cancer, and may be associated with any lung cancer of any cell type. It is most frequently associated with squamous and adenocarcinoma and least frequently associated with small cell carcinoma.[26,31] In a previous study on 111 patients with pathologically proven lung cancer, clubbing was present in 29%, women were more affected than men (40% *vs.* 19%) and clubbing was more common in nonsmall cell lung cancer than small cell lung cancer (35% *vs.* 4%).[29,31] In our study a higher frequency of clubbing was present (40%) and in contrast to the previous study, men were more commonly affected than women (89% *vs.* 11%); however, we report similar findings of clubbing being more common in non-small cell lung cancer than small cell lung cancer (87% *vs.* 17).[26,29] Chest radiograph plays a critical role in diagnosing lung cancer. Most of the lung tumors are detected on chest radiograph, but unfortunately the majority of patients have advanced stage at presentation.[32–34] In our study, the right lung was affected more frequently than the left (65% *vs.* 38%) respectively. Furthermore, the right and left upper lobes had the highest incidence of lung cancer (60% *vs.* 74%) radiologically as compared with middle (22%, 19%) and lower lobes (27%, 28%) respectively. It was not clear why the upper lobes were more susceptible to lung cancer than other lobes. However, it is well known that upper lobes are less vascular, and more aerated than the lower ones, and more affected by smoking, therefore, we question whether some or all of these factors may have an effect on the distribution of the lung cancer among affected patients. In this study, lung mass, atelectasis/consolidation and pleural effusion were the most common abnormalities found as detected by chest radiographs (69%, 50%, and 35%) result that rose to (83%, 61%, and 36%) by CT scan of the chest respectively. Furthermore, CT scan of the chest is more sensitive than chest radiographs in detecting early stage of lung cancer and in assessing the pattern of distribution of affected lymph nodes in patients with lung cancer. Mediastinal lymph nodes have the highest incidence of involvement in lung cancer (51%) followed by the hilar (30%) abnormalities that can't be assessed clearly in chest radiographs.[34–36] In this study, lung cancer had metastases to the bones and mediastinal lymph nodes in almost 50% the patients at presentation. Bone pain is present in up to 25% of all patients at presentation.[26,28,29] In our patients, bone pain was far less common (11%) than reported in the literature although high in incidence of bone metastases at presentation (49%). Liver metastases occur commonly with lung cancer, and usually carry poor prognosis.[26,28,29] Almost 30% of our patients had liver metastases and 14% had jaundice at presentation. Adrenal metastases may rarely occur and are commonly seen in small cell lung cancer and mainly discovered during staging. Their presence reflects extensive disease.[37] In our patients, adrenal metastases occurred in 9% at presentation which was slightly higher than the 7% reported in the literature. Brain metastases occur in 10% of patients at presentation. Lung cancer is a primary site of approximately 70% of symptomatic brain metastases.[38] However, brain metastases were rarely seen in our patients (4%). FFB is used to diagnose both central and peripheral lung lesions. It is the simplest method for obtaining material from the suspicious lesion with little morbidity and almost negligible mortality.[39,40] The overall diagnostic yield of bronchoscopy for central lung cancer is about 70% and increases to 90% when the tumor is visible bronchoscopically.[40] In our study, 60% of lung cancer was diagnosed by FFB. The overall yield of bronchoscopy was high and reached up to 80%, as most of the lung cancer in our patients was advanced at presentation. These findings were similar to those reported in the literature.[39,40] In our study, the most common sites of abnormal findings observed (n = 68) were in the right and left upper bronchi (24% *vs.* 16%) followed by right middle bronchus and lingula (12% for each) while the least affected sites were the right and left lower bronchi (9% *vs.* 6%). The reported pattern of distribution of lung cancer in which upper lobes were affected more than middle ones and the middle ones were affected more than lower ones was a very interesting observation not previously reported. CT guided transthoracic needle aspiration (TTNA) is the procedure of choice for sampling peripheral lung lesion with a diagnostic accuracy of 80–95%.[37,38] In our patients, 34% were diagnosed with CT-guided true cut biopsy, and similar to the literature, the diagnostic yield was 91%. The four major histologic subtypes of lung cancer include squamous cell carcinoma (25%), adenocarcinoma (30%), large cell carcinoma (10%), and small cell carcinoma (20%).[23,24,26,44] In the past decade, adenocarcinoma has surpassed squamous cell carcinoma as the most common histologic subtype of lung cancer in the US.[43,44] In KSA, a recent report showed similar incidences for adenocarcinoma (30%) and squamous cell carcinoma (27%) while a lower incidence was seen for large cell carcinoma (6%), and small cell carcinoma (10%).[7] Contrary to previous reports,[7,23,24,44,45] squamous cell carcinoma has the highest incidence among our cases (52%), while adenocarcinoma (27%), large cell carcinoma (6%), and small cell carcinoma (15%) have almost similar incidences [Figure 1]. The higher incidence of squamous cell was probably related to the higher incidence of cigarette smoking that reached up to 85% in those patients. Interestingly, 40% of squamous cell type occurred in those who smoked 20-36 pack/yr, while 52 % occurred in those who smoked >36 pack/yr. Furthermore, 86% of squamous cell type occurred in those who smoked >20 years. In adenocarcinoma; 61% of our patients were nonsmokers, but lung cancer occurred mainly in those who smoked for >20 pack/yr for >20 years ((92% for each). In small cell type, most of the patients were smokers (82%) and similar to the other cell types, lung cancer occurred in 79% of those who smoked >20 pack/yr and in 93% of those who smoked >20 years. Large cell cancer occurred in 71% of our patient particularly in those who smoked >20 pack/day for >20 years. These findings show clearly that the incidence of lung cancer sharply increased with the number of pack/yr smoking (20 pack/yr or more) and with the duration of smoking (20 yr or more) irrespective of the cell type of lung cancer [Figure 2]. In this study, the association of lung cancer and shisha (hubbly-bubbly) was observed in 2 patients who smoked 3 to 5 shisha per day for almost 20 years. One patient was female aged 44 yr and had squamous cell carcinoma while the other patient was male aged 62 yr and had adenocarcinoma. We belief this is the first study to report an association between lung cancer and shisha; previous studies showed that shisha smoking can cause lip cancer and increased chronic respiratory symptoms.[46,47]. Further studies with large sample of shisha smokers are necessary to assess whether shisha, like cigarettes, can cause lung cancer. Hypercalcemia is a common endocrine paraneoplastic syndrome associated with lung cancer. It is frequently secondary to bone metastasis or due to production of a parathyroid hormone-related peptide and commonly associated with squamous cell carcinoma.[48] In our study, 13% of patients with lung cancer have hypercalcemia mainly associated with squamous cell type [Figure 3]. Hyponatremia may occur in lung cancer due to either increase production of antidiuretic hormone (ADH), syndrome of inappropriate ADH (SIADH), or atrial natriuretic hormone. Excess ADH production can be documented in up to 70% of patients with lung cancer while SIADH is less common.[49] SAIDH is mainly associated with small cell lung cancer, although other malignant tumors of the lung may rarely be associated with this syndrome.[29,31] In our study, hyponatremia was present in 56% of patients that may be due to increase production of ADH; however, SIADH was rarely seen. Furthermore, contrary to the literature,[29] hyponatremia was mainly associated with adenocarcinoma and to a lesser extent with small cell carcinoma [Figure 4]. In conclusion, the majority of our patients had an advanced stage of lung cancer at presentation. Squamous cell carcinoma and adenocarcinoma were the most common cell types. Duration of smoking and the number of pack/yr were the two major risk factors associated with a high incidence of lung cancer. The lack of effective screening tests for the early detection of lung cancer has made prevention or cessation of smoking of utmost importance to reduce the risk of lung cancer.
